# Challenges associated with dapsone for leprosy treatment in Indonesia - urgent need for access to alternative antimicrobial drugs

**DOI:** 10.1016/j.lansea.2025.100555

**Published:** 2025-02-26

**Authors:** Hana Krismawati, Maria Harianja, Antonius Oktavian, Claus Bøgh, Messe R. Ataupah, Ruth D. Laiskodat, Arry Pongtiku, Annemieke Geluk, J. Kevin Baird, Raph L. Hamers, Hardyanto Soebono, Stephen L. Walker, Marlous L. Grijsen

**Affiliations:** a Center of Health System and Strategy, Ministry of Health, Jakarta, Indonesia; b Sumba Foundation, Sumba, Indonesia; c Regional Public Health Laboratory of Papua, Ministry of Health, Papua, Indonesia; d Province Health Office, East Nusa Tenggara, Indonesia; e Province Health Office, Papua, Indonesia; f Leiden University Center of Infectious Diseases (LUCID), Leiden, the Netherlands; g Oxford University Clinical Research Unit Indonesia, Faculty of Medicine Universitas Indonesia, Jakarta, Indonesia; h Centre for Tropical Medicine and Global Health, Nuffield Department of Medicine, University of Oxford, UK; i Department of Dermatology and Venereology, Universitas Gadjah Mada, Yogyakarta, Indonesia; j Center for Tropical Medicine, Faculty of Medicine, Public Health and Nursing, Universitas Gadjah Mada, Yogyakarta, Indonesia; k Clinical Research Department, London School of Hygiene and Tropical Medicine, London, UK; l Hospital for Tropical Diseases, University College London Hospital NHS Foundation Trust, London, UK

**Keywords:** Acute hemolytic anemia, Dapsone, Dapsone hypersensitivity syndrome, G6PD deficiency, HLA-B13:01, Indonesia, Leprosy, Low- and middle-income countries, Morbus hansen, Multi-drug therapy, Pharmacogenetics

## Abstract

Leprosy is effectively treated with multi-drug therapy (MDT), a regimen containing three antibiotic drugs, including dapsone - a sulfone drug associated with potentially life-threatening adverse drug reactions. Specifically, dapsone hypersensitivity syndrome (DHS), linked to HLA-B∗13:01 polymorphism, and hemolytic anemia associated with glucose-6-phosphate dehydrogenase deficiency (G6PDd).

Both of these pharmacogenetic polymorphisms can be prevented through diagnostic screening before MDT initiation averting potential complications. However, in leprosy-endemic areas like Indonesia, access to these tests often remains inaccessible due to high costs and limited laboratory capacity. Additionally, alternative dapsone-sparing treatment regimens are usually unavailable or unaffordable, restraining individuals onto suboptimal dual-therapy with rifampicin and clofazimine, which has uncertain efficacy. We raise concerns regarding the safety of dapsone-containing MDT without routine pharmacogenetic screening and the unavailability of alternative regimens. We call for action to address persisting global health inequities in care delivery, ensuring all individuals receive the safest and most effective leprosy treatment options.

Leprosy is a highly stigmatized neglected tropical skin disease (skin-NTD) caused by *Mycobacterium leprae* and, to a lesser extent, by *Mycobacterium lepromatosis*.^1^ It affects more than 200,000 people every year, particularly in low- and middle-income countries (LMIC), and is treated with antimicrobial multi-drug therapy (MDT), including rifampicin, clofazimine, and dapsone, as recommended by the World Health Organization (WHO).^2^ MDT is administered orally for a duration of six or twelve months depending on the type of leprosy. Observational studies conducted in Bangladesh and Brazil, with over a decade follow-up, have shown high cure rates of MDT.^3^^,^^4^ Since its introduction in 1982, MDT has been provided free of charge and has been administered globally to ∼20 million people affected by leprosy.^5^

Dapsone is a sulfone drug with bacteriostatic and anti-inflammatory properties. It inhibits the synthesis of bacterial folic acid and reduces neutrophil and eosinophil chemotaxis, as well as reactive oxygen-species (ROS) production.^6^ Dapsone was introduced as monotherapy for leprosy in the mid-20th century. However, prolonged monotherapy resulted in the emergence of dapsone resistance, prompting the introduction of MDT in 1982 as a more effective treatment strategy. Dapsone is also used to treat dermatitis herpetiformis, linear IgA dermatosis, *Pneumocystis jirovecii* pneumonia, and toxoplasmosis.^6^^,^^7^

The effectiveness of MDT for leprosy has, however, overshadowed the associated adverse drug reactions (ADR).^8^ Overall, there is lack of comprehensive and systematic data on MDT-related ADR, primarily due to the retrospective nature and limited scope of most studies. ADR related to dapsone include gastro-intestinal symptoms, hemolytic anemia, methemoglobinemia, hepatitis, bone marrow aplasia, and dapsone hypersensitivity syndrome (DHS).^8^, ^9^, ^10^, ^11^ They can be severe and pose significant challenges, especially in leprosy endemic-communities in LMIC, where certain pharmacogenetic predispositions are more common. Specifically, the human leukocyte antigen (HLA)-B∗13:01 allele linked to DHS, and glucose-6-phosphate dehydrogenase deficiency (G6PDd), which is a risk factor for acute hemolytic anemia.^12^^,^^13^

Here we review the available data on the dapsone-related ADR associated with HLA-B∗13:01 and G6PDd, and the resultant real-world challenges in the clinical management of leprosy among affected populations in resource-constrained settings. We present supporting data from the literature, including preliminary data from our ongoing projects in eastern Indonesia. Indonesia reported 14,000 new cases in 2023, the third highest number after India and Brazil.^14^

## Dapsone hypersensitivity syndrome

Initially described as ‘dapsone syndrome’ in 1949 in Nigeria, DHS is the most serious, life-threatening dapsone-related ADR and one of the main causes of death in individuals affected by leprosy.^15^ DHS is characterized by fever, skin rash, hepatitis, and pulmonary and other systemic manifestations, which usually develop within six weeks of dapsone initiation.^13^ DHS may lead to irreversible organ damage if not recognized promptly, and has an estimated mortality rate of 9.9–12.5%.^12^^,^^16^ The estimated global prevalence of DHS in persons exposed to dapsone is 1.4% (95% CI 1.2–1.7%),^16^ showing marked variability across different populations, with most reported cases originating from Asia. A genome-wide association study in China involving persons with leprosy who had developed DHS, was the first to identify a strong association with HLA-B∗13:01 (OR 20.5; *p* < 0.001),^17^ which was later confirmed in other Asian populations,^18^, ^19^, ^20^, ^21^ with reported population frequencies of HLA-B∗13:01 between 1 and 21%.^17^^,^^22^ Prospective HLA-B∗13:01 screening and subsequent exclusion of dapsone from MDT for individuals with HLA-B∗13:01, reduced the incidence of DHS to zero in a Chinese cohort study.^22^

The prevalence of DHS in Indonesia is unknown, and is likely to vary due to the population's genetic diversity. A local surveillance report from Papua province described an annual DHS incidence of 11% in individuals with leprosy treated with WHO-MDT^23^ and was strongly linked to the presence of HLA-B∗13:01 allele.^19^ It was estimated that 10 persons need to be screened for HLA-B∗13:01 to prevent one case of DHS.^19^ Participants screened for inclusion in the MetLep trial in Papua province, a treatment trial of adjunctive metformin combined with MDT for multibacillary leprosy (NCT05243654), are tested for HLA-B∗13:01 prior to MDT initiation using the commercial qPCR kit from Nalagenetics^24^^,^^25^; 20 of 70 (29%) MetLep participants enrolled to date tested positive for the HLA-B∗13:01 allele (*unpublished data*). For these individuals, dapsone was removed from the MDT in accordance with WHO guidelines.^26^

## Glucose-6-phosphate dehydrogenase deficiency

Individuals deficient in G6PD are at increased risk of developing potentially severe and life threatening acute hemolytic anemia, which may be triggered by infections, foods or drugs, including sulfones like dapsone, primaquine (for radical cure of *P vivax* malaria), and others.^27^ G6PDd is inherited in an X-linked fashion affecting an estimated 500 million people worldwide. The distribution of G6PDd closely relates with regions where malaria is or has been endemic, as the deficiency imparts a selective advantage against malaria infection.^28^ The prevalence of G6PDd is around 8% of the general population in these countries,^27^ and highest in Africa, Southeast Asia, the Mediterranean and the Middle East. In a recent case series in Sumba, eastern Indonesia,^29^ individuals diagnosed with leprosy were tested for G6PDd using the STANDARD biosensor point-of-care semi-quantitative analyzer (SD Biosensor, South Korea) prior to MDT initiation. Seven of 70 individuals (10%) were G6PD deficient (*unpublished data*), and dapsone was excluded from MDT. That proportion was consistent with findings from large surveys conducted among the general population in eastern Indonesia.^30^^,^^31^

## Poor access to diagnostics and alternative treatment regimens in leprosy-endemic areas

In many endemic areas, access to diagnostic tests for HLA-B∗13:01 and G6PD is challenging due to their high costs, estimated between USD 10 and 12 per test, and limited laboratory capacity. Most studies reporting on dapsone-related ADR, as part of MDT, have not performed pharmacogenetic screening. Treatment modifications or discontinuations due to dapsone-related ADR, primarily hemolytic anemia, is common among individuals receiving MDT, reported for 25% (48/194) of people in a study in Brazil^11^^,^^32^ and 17% (26/150) in India,^9^ settings in which anemia is further complicated by co-infections and malnutrition. In the Uniform-MDT trial in Brazil, 24 out of 753 participants (3.2%) stopped dapsone because of ADR and received alternative treatment.^33^ A study in Nepal revealed that 4 out of 18 leprosy-affected individuals who experienced dapsone-related ADR, died from severe anemia and DHS.^10^

In many LMIC, alternative antimicrobial regimens that exclude dapsone, typically containing three of the following drugs: rifampicin, rifapentine, ofloxacin, clarithromycin, minocycline and/or moxifloxacin,^34^, ^35^, ^36^ are often not available nor affordable for patients. Consequently, the only option available to affected individuals is the continuation of dual-therapy with rifampicin and clofazimine, as recommended by WHO guidelines.^26^ There is, however, a lack of comprehensive data from prospective studies on the long-term clinical effectiveness of this pragmatic approach in terms of relapse rates, occurrence of leprosy reactions and the development of drug resistance. The latter is further complicated by the limited understanding of clofazimine's mechanism of action and the genetic markers linked to resistance.^37^ A retrospective study in Nepal of 60 leprosy-affected individuals, treated with dual-therapy with rifampicin and clofazimine due to dapsone-related ADR, showed a consistent decrease in the mean bacillary index of those who underwent repeat slit-skin smear testing. No relapses were reported, although the follow-up period was insufficient to assess long-term effectiveness.^38^

Based on the limited available data globally and our own experience in eastern Indonesia, we raise concern about the safety of prescribing dapsone in the absence of routine pharmacogenetic screening and accessible alternative drug regimens. To address health disparities in care delivery for underserved populations and to achieve the WHO strategic goal ‘Towards Zero Leprosy’ for 2021–2030, we propose four actions to mitigate the risk of harm.

First, to ensure early detection and management of any potential ADR, it is important that all individuals newly diagnosed with leprosy receive a full blood count evaluation prior to MDT initiation and 4 and 8 weeks post-treatment initiation. Furthermore, thorough counseling on potential ADR associated with MDT should be provided, including educating people affected by leprosy (and where appropriate their carers) of warning signs and necessary actions to take if an ADR is suspected.^13^ Healthcare providers play a vital role in this process and must receive comprehensive training to deliver clear and accurate information, effectively monitor patients, and promptly identify and manage ADR. A recent study developed a prediction tool to assist healthcare professionals to estimate which MDT-treated individuals were at risk of dapsone-related ADR; the identified predictors were multibacillary leprosy, female sex and higher education, although the latter is likely confounded by a higher awareness for ADR among higher educated people.^39^ A limitation of the prediction model was that it did not incorporate any laboratory or genetic tests results, which reduces its usefulness in populations with a high frequency of HLA-B∗13:01 and/or G6PDd.

Second, these present knowledge gaps highlight the need for well-designed prospective studies to better estimate the frequency and severity of MDT-related ADR, subsequent need for drug modification or discontinuation, and their impacts on clinical outcomes in terms of cure or relapse rates, drug resistance, reactions, and mortality.

Third, our observations among populations in eastern Indonesia suggest that HLA-B∗13:01 and G6PDd polymorphisms are common, exposing individuals to potential dapsone-related ADR. Further evidence is required on the clinical benefits and cost-effectiveness of personalized treatment strategies based on universal pharmacogenetic testing prior to MDT initiation across various geographical areas. Such recommendation would follow similar existing guidance for other well-known HLA polymorphisms associated with severe ADR. For example, HLA-B∗15:02 linked to carbamazepine-induced Stevens-Johnson syndrome and toxic epidermal necrolysis in Southeast Asian populations, and HLA-B∗57:01 linked with abacavir hypersensitivity syndrome in persons living with HIV.^40^

Finally, well-designed clinical trials are fundamental to evaluate the efficacy, tolerability, safety and cost-effectiveness of alternative antimicrobial regimens for individuals with leprosy at high risk of dapsone-related ADR, potentially averting the need for individual genetic testing.^34^^,^^35^^,^^41^ Furthermore, there is a pressing need to explore novel treatment regimens that offer shorter treatment durations and enhanced drug adherence. Antituberculosis drugs like bedaquiline, a diarylquinoline with a long half-life, and telacebec, a QcrB inhibitor, have the potential to revolutionize the management of leprosy.^42^^,^^43^

In conclusion, the high occurrence of the HLA-B∗13:01 allele and G6PDd among individuals affected by leprosy in Indonesia, raises concerns about the safety of dapsone-containing MDT in the absence of routine pharmacogenetic screening and the poor accessibility to alternative antibiotic regimens that have more favorable ADR profiles. There is an urgent need for evidence on personalized treatment strategies to ensure all individuals receive the safest and most effective care, which is essential for advancing health equity globally and reducing the burden of leprosy-related morbidity.

## Contributors

All authors have contributed equally to this work.

## Declaration of interests

The MetLep trial is funded by the Leprosy Research Initiative and Turing Foundation [FP20.4/708.20.04]. The skin care programme in Sumba was supported by a grant from the L'Oreal International Award for Social Responsibility in Dermatology and GLODERM x CeraVe Access Grant. Marlous Grijsen, Raph Hamers and Kevin Baird are supported by the Wellcome Africa Asia Programme Vietnam core grant (106680/Z/14/Z). The authors declare that they have no conflicts of interests to report.
